# Focusing on fidelity: narrative review and recommendations for improving intervention fidelity within trials of health behaviour change interventions

**DOI:** 10.1080/21642850.2020.1738935

**Published:** 2020-03-12

**Authors:** E. Toomey, W. Hardeman, N. Hankonen, M. Byrne, J. McSharry, K. Matvienko-Sikar, F. Lorencatto

**Affiliations:** a Health Behaviour Change Research Group, School of Psychology, National University of Ireland Galway, Galway, Ireland; b Health Promotion Research Group, School of Health Sciences, University of East Anglia, Norwich, UK; c Faculty of Social Sciences, University of Helsinki, Helsinki, Finland; d School of Public Health, University College Cork, Cork, Ireland; e Centre for Behaviour Change, University College London, London, UK

**Keywords:** Health behaviour change, intervention fidelity, complex interventions, trial methodology, implementation science

## Abstract

**Background:** Interventions to change behaviour have substantial potential to impact positively on individual and overall public health. Despite an increasing focus on health behaviour change intervention research, interventions do not always have the desired effect on outcomes, while others have diluted effects once implemented into real-life settings. There is little investment into understanding *how* or *why* such interventions work or do not work. Methodological inadequacies of trials of behavioural interventions have been previously suggested as a barrier to the quality and advancement of behavioural research, with intervention fidelity acknowledged as a key area for improvement. However, there is much ambiguity regarding the terminology and conceptualisation of intervention fidelity and a lack of practical guidance regarding how to address it sufficiently, particularly within trials of complex behavioural interventions.

**Objectives:** This article outlines specific issues concerning intervention fidelity within trials of health behaviour change interventions and suggests practical considerations and specific recommendations for researchers, with examples from the literature presented.

**Conclusions:** Recommendations pertain to (1) clarifying how fidelity is defined and conceptualised, (2) considering fidelity beyond intervention delivery, (3) considering strategies to both enhance and assess fidelity, (4) making use of existing frameworks and guidance, (5) considering the quality and comprehensiveness of fidelity assessment strategies, (6) considering the balance between fidelity and adaptation and (7) reporting the use of fidelity enhancement and assessment strategies and their results. Suggestions for future research to improve our understanding of, and ability to, address fidelity in behaviour change interventions are also provided.

## Health behaviour change research – a growing field

It is well recognised that health behaviours such as smoking, diet and physical activity play a crucial role in influencing individual and population-level health and well-being outcomes. Accordingly, health behaviour change is a growing focus of research, with estimates of hundreds of trials of behaviour change interventions conducted each day globally. Despite this increasing focus, many of these trials have modest and variable effects (Flodgren, Gonçalves-Bradley, & Summerbell, 2017), and evidence for long-term impact of such interventions is lacking (Jepson, Harris, Platt, & Tannahill, 2010; Kwasnicka, Dombrowski, White, & Sniehotta, 2016). Furthermore, the uptake of existing effective behavioural interventions into practice and policy settings has been limited (Bacon et al., 2014), and interventions that are shown to be effective in research settings often have diluted effects once implemented into real-life settings (Feldman, Silapaswan, Schaefer, & Schermele, 2014). Concerns about the methodological inadequacies of trials of behavioural interventions have been raised as a barrier to the quality and advancement of behavioural research, and subsequently to the implementation of these interventions into policy and practice (Bacon et al., 2014). Amongst these methodological inadequacies, intervention fidelity has been acknowledged as a key area for improvement and future research. In this paper, we discuss several commonly highlighted methodological shortcomings relating to intervention fidelity, provide recommendations for improving intervention fidelity in behavioural trials based on specific examples, and suggest areas for future research.

Within behavioural research, intervention fidelity refers to the ‘methodological strategies used to monitor and enhance the reliability and validity of behavioural interventions’ (Bellg et al., 2004). In essence, intervention fidelity is the degree to which interventions are put into practice as intended (Carroll et al., 2007), and is of paramount importance for health behaviour change research. Without adequate assessment and evaluation of intervention fidelity, one cannot be certain that any intervention effects observed in trials are due to the intervention being investigated and not due to variability in how it was actually put into practice (Borrelli, 2011). At the same time, ensuring that behaviour change interventions are delivered and received with good fidelity maximises the efficiency of research and potential impact, by ensuring that trial evaluations adequately and accurately test their hypotheses (Carroll et al., 2007; Johnson-Kozlow et al., 2008). For example, significant results may be due to an effective intervention or due to unknown elements added to the intervention, leading to rejection of the null hypothesis when it is true (Type I error). Alternatively, non-significant results may be due to an ineffective intervention or essential elements omitted from the intervention, leading to an acceptance of the null hypothesis when it is false (Type II error) (Banerjee, Chitnis, Jadhav, Bhawalkar, & Chaudhury, 2009). In addition to enabling an accurate interpretation of an intervention’s results, assessing fidelity can also help identify the essential features or elements of the intervention and enhance the ability to accurately replicate effective behavioural interventions (Robb, Burns, Docherty, & Haase, 2011). Furthermore, by assessing intervention fidelity comprehensively, understanding can be gained about what adaptations may have been made during the trial, and how these may have affected outcomes. This can also guide refinement of the intervention, and provide information about the feasibility of further dissemination and implementation of the intervention into other settings beyond the trial (Mowbray, Holter, Teague, & Bybee, 2003).

Beyond trials of effectiveness of behavioural interventions, addressing intervention fidelity is also important across all stages of developing, evaluating, and scaling up or implementing such interventions into policy or practice (Craig et al., 2008). For example, considering intervention fidelity during the initial stages of intervention development is important to help specify the core components of an intervention and link to theories of change (Abry, Hulleman, & Rimm-Kaufman, 2014). Addressing intervention fidelity within feasibility studies can help explore the feasibility of strategies to both enhance (e.g. treatment manuals or reminder prompts) and assess (e.g. direct observations or checklists) fidelity within the main trial (Toomey, Matthews, Guerin, & Hurley, 2016). Assessing intervention fidelity at the feasibility stage can also provide important information, such as the identification of intervention elements with lower than expected fidelity, which can be used to optimise the intervention prior to the fully powered trial (Hankonen et al., 2017). For evaluating the implementation of behavioural interventions into real-world contexts, it is also important to consider fidelity in terms of enhancing and assessing fidelity to the original intervention to minimise dilution of effects while achieving a balancing to enable appropriate adaptation for local context (McCrabb et al., 2019; McHugh, Murray, & Barlow, 2009), as well as the fidelity of intended implementation strategies (Slaughter, Hill, & Snelgrove-Clarke, 2015). The focus in this paper is predominantly on addressing intervention fidelity within outcome evaluations of behavioural interventions (e.g. trials of effectiveness); however, much of this is highly relevant for feasibility and implementation studies also, and implications for both will be highlighted throughout the manuscript.

Despite its importance, traditionally there has been substantial investment of resources into evaluating the effectiveness and cost-effectiveness of behaviour change interventions, with comparatively less investment into intervention fidelity and understanding *how* or *why* the interventions work or do not work (Moore et al., 2015). In an attempt to address this, in 2015 the UK Medical Research Council (MRC) published guidance for conducting process evaluations of complex interventions, with intervention fidelity acknowledged as one of the key elements of process evaluations (Moore et al., 2015). However, due to the substantial breadth of this guidance, it did not provide recommendations about how to address intervention fidelity specifically, but acknowledged fidelity as an important area requiring further attention (Moore et al., 2015). Provision of in-depth and practical considerations regarding how to address fidelity sufficiently, particularly within trials of complex health behaviour change interventions, is therefore warranted.

## Issues with intervention fidelity – what is not being done, and when it is being done, what is lacking?

A common finding of systematic reviews across multiple areas of health behaviour change is that intervention fidelity is often poorly addressed in trials of behaviour change interventions (Table 1). In particular, these reviews often identify several key methodological issues regarding how fidelity is currently being addressed. In an attempt to explore these issues further, McGee et al. conducted an international survey in 2018 amongst 264 researchers and healthcare professionals with trial experience of healthcare behaviour change interventions to explore the knowledge, practice (use of intervention fidelity strategies), barriers and facilitators to practice and attitudes towards addressing intervention fidelity (Mcgee, Lorencatto, Matvienko-Sikar, & Toomey, 2018). The survey found that despite a good awareness of the importance of intervention fidelity by 89.7% of participants, poor knowledge and understanding was the most commonly cited barrier to intervention fidelity by 77.4%. The survey also identified inconsistent terminology and definitions amongst the most frequently identified barriers. In the following section, we outline each of the issues highlighted across existing systematic reviews and further evidenced within the McGee et al. survey. Specifically, these include a lack of standardisation regarding fidelity concepts and terminology, a limited focus on intervention fidelity beyond delivery, poor use of existing fidelity frameworks or guidance, a lack of focus on the methodological quality of assessment methods, a lack of focus on the balance between fidelity and adaptation and substandard reporting.

**Table 1.** Commonly identified methodological issues regarding intervention fidelity in literature reviews.

Issue identified	Literature reviews across multiple behaviour change research topics where issue mentioned (review reference)
1. Lack of standardisation regarding how fidelity is conceptualised and defined	Clinical psychology, behaviour therapy (Moncher & Prinz, 1991) Clinical psychology, school-based prevention (Dane & Schneider, 1998) Substance-abuse prevention, school-based settings (Dusenbury, Brannigan, Falco, & Hansen, 2003) Health behaviour change interventions (Borrelli et al., 2005) Diabetes self-management interventions (Schinckus, Van Den Broucke, & Housiaux, 2014) Healthcare provider behaviour change interventions (Slaughter et al., 2015) Psychosocial interventions (Prowse & Nagel, 2015) Health behaviour change interventions (Rixon et al., 2016) Physiotherapy behaviour change interventions (O’Shea, Mccormick, Bradley, & O’Neill, 2016) Physical activity behaviour change (Lambert et al., 2017)
2. Limited focus beyond assessing fidelity of delivery	Clinical psychology, behaviour therapy (Moncher & Prinz, 1991) Mental health, child and adolescent psychosocial interventions (McArthur, Riosa, & Preyde, 2012) Mental health parent training interventions (Garbacz, Brown, Spee, Polo, & Budd, 2014) Healthcare provider behaviour change interventions (Slaughter et al., 2015) Behavioural paediatric obesity interventions (JaKa et al., 2016) Health behaviour change interventions (Rixon et al., 2016) Physiotherapy behaviour change interventions (O’Shea et al., 2016) Physical activity behaviour change (Lambert et al., 2017) Infant feeding interventions, childhood obesity (Toomey et al., 2018)
3. Limited use of existing fidelity frameworks or guidance	Diabetes self-management interventions (Schinckus et al., 2014) Healthcare provider behaviour change interventions (Slaughter et al., 2015) Psychosocial interventions (Prowse & Nagel, 2015) Physiotherapy behaviour change interventions (O’Shea et al., 2016) Physical activity behaviour change (Lambert et al., 2017) Face-to-face health behaviour change interventions (Walton, Spector, Tombor, & Michie, 2017)
4. Lack of focus on quality and comprehensiveness of fidelity assessment strategies	Clinical psychology, behaviour therapy (Moncher & Prinz, 1991) Clinical psychology, school-based prevention (Dane & Schneider, 1998) Substance-abuse prevention, school-based settings (Dusenbury et al., 2003) Substance-abuse treatment and prevention (Baer et al., 2007) Occupational therapy, sensory integration interventions (Parham et al., 2007) Diabetes self-management interventions (Schinckus et al., 2014) Health behaviour change interventions (Rixon et al., 2016) Physical activity behaviour change (Lambert et al., 2017) Face-to-face health behaviour change interventions (Walton et al., 2017)
5. Lack of explicit focus on the balance between fidelity and adaptation	Clinical psychology, school-based prevention (Dane & Schneider, 1998) Substance-abuse prevention, school-based settings (Dusenbury et al., 2003)
6. Poor reporting of how intervention fidelity is addressed	Clinical psychology, behaviour therapy (Moncher & Prinz, 1991) Substance-abuse prevention, school-based settings (Dusenbury et al., 2003) Occupational therapy, sensory integration interventions (Parham et al., 2007) Self-management interventions, chronic pain (Toomey, Currie-Murphy, Matthews, & Hurley, 2014) Diabetes self-management interventions (Schinckus et al., 2014) Healthcare provider behaviour change interventions (Slaughter et al., 2015) Behavioural paediatric obesity interventions (JaKa et al., 2016) Physiotherapy behaviour change interventions (O’Shea et al., 2016) Infant feeding interventions, childhood obesity (Toomey et al., 2018)

### Issue 1: Lack of standardisation regarding how fidelity is conceptualised and defined

Within the fidelity literature, numerous terms exist that have often been used interchangeably. These include treatment fidelity, treatment integrity, intervention fidelity, implementation fidelity, programme fidelity, programme integrity, procedural reliability and therapist adherence/competence (Di Rezze, 2012; Montgomery, Underhill, Gardner, Operario, & Mayo-Wilson, 2013). This makes it difficult to understand how fidelity is conceptualised and defined.

Inconsistencies in nomenclature are somewhat illuminated by tracing the historical development of fidelity literature. When the concept of fidelity first emerged in the psychotherapy literature in the 1970s and 1980s, the term ‘treatment fidelity’ appeared to be synonymous with ‘treatment integrity’: both were defined as ‘treatment delivered as intended’, focusing on the delivery of a treatment or intervention by the provider (Quay, 1977; Yeaton & Sechrest, 1981). As the concept continued to develop, fidelity evolved to include ‘treatment differentiation’, i.e. considering fidelity of intervention delivery within both experimental and comparator intervention conditions within a trial (Moncher & Prinz, 1991), and therapist competence or the ‘skill with which the treatment is delivered’ (Waltz, Addis, Koerner, & Jacobson, 1993). Other researchers including Lichstein, Riedel, and Grieve (1994) and the National Institutes for Health Behaviour Change Consortium (NIHBCC) then added the concepts of treatment receipt (participants’ understanding and demonstration of ability to use skills learned) and treatment enactment (participants’ actual use of skills learned in daily life), including the intervention participant as an active part of the fidelity process (Bellg et al., 2004; Lichstein et al., 1994). Researchers also incorporated the study design or theoretical underpinning and intended causal mechanisms as an aspect to be addressed within fidelity (Bellg et al., 2004; Gearing et al., 2011; Montgomery et al., 2013). Thus, fidelity evolved into a broader multi-faceted concept, encompassing more than just the fidelity of intervention delivery by the provider, to include the fidelity of each stage of an intervention where an action was intended to occur, or where an assumption might be made about what actually happened (Prowse & Nagel, 2015). This evolution over time means that different definitions and concepts exist within the literature and are used inconsistently and interchangeably as outlined previously.

### Recommendation 1: Clarify how fidelity is defined and conceptualised

Without clear consensus on the most appropriate or acceptable definitions for fidelity, the most practical recommendation to advance the science in the interim is to ensure clarity and explicitness in how fidelity is defined and conceptualised from the outset in each study. This includes outlining how and why fidelity data will be used. For example, the process evaluation of the AFFINITIE (Development & Evaluation of *A*udit and *F*eedback *IN*terventions to *I*ncrease evidence-based *T*ransfusion pract*I*c*E*) trial includes a multidimensional assessment of fidelity for two audit and feedback interventions to assess the extent to which fidelity contributes to observed outcomes (i.e. proportion of unnecessary transfusions) (Lorencatto et al., 2016). This process evaluation protocol provides and references an explicit definition of intervention fidelity, and describes how fidelity will be addressed with reference to the NIHBCC framework (Borrelli et al., 2005). The protocol also details how contextual influences that might influence responses by clinical staff to the feedback interventions will be explored, making reference to the MRC process evaluation guidance (Moore et al., 2015). To facilitate further improvements in this area, future fidelity research could aim to synthesise existing terminology and concepts and use stakeholder consensus to build towards a common language e.g. by conducting a structured concept analysis of intervention fidelity, followed by a consensus-building Delphi-type study.

### Issue 2: Limited focus beyond assessing fidelity of delivery

Despite the evolution of fidelity into a broader multidimensional concept, many still view fidelity in its original capacity, defining it as ‘the *delivery* of the intervention or treatment as intended’ (Breitenstein et al., 2010; Prowse & Nagel, 2015). For example, treatment delivery is often the most measured and reported upon component of fidelity (Garbacz et al., 2014; Gearing et al., 2011; Lambert et al., 2017; O’Shea et al., 2016), and was the most commonly identified component of fidelity by 95.7% of surveyed researchers and healthcare professionals with experience of trials of complex healthcare interventions (McGee et al., 2018). Moreover, fidelity of treatment delivery is often only viewed as relevant within the experimental condition (McGee et al., 2018). This is problematic as recent research identified substantial variability and complexity within the active content of comparator groups in behavioural trials, and that this content is often under-reported (De Bruin et al., 2020). As such, adequately quantifying the intervention content within both experimental and comparator conditions is vital to facilitate a comprehensive approach to evaluating intervention fidelity within behavioural trials.

Others also debate the addition of fidelity components beyond treatment delivery such as treatment receipt and enactment, arguing that participant engagement with the intervention and use of intervention skills in daily life relates to treatment adherence and intervention effectiveness and not to intervention fidelity (Gearing et al., 2011). For example, treatment enactment was the least frequently endorsed component in the same survey, selected by only 13.6% of participants as being part of intervention fidelity (McGee et al., 2018). However, treatment receipt is defined as incorporating more than just adherence, but also includes participant understanding (Bellg et al., 2004). Increasingly, it is accepted that patients are ‘active participants’ of health behaviour change interventions rather than ‘passive recipients’ (Rixon et al., 2016). Previous research has shown that enactment of skills taught in the intervention relates to intervention effectiveness on behavioural outcomes (Hankonen et al., 2015; Köykkä et al., 2019). As such, assuming that both experimental and comparator group participants will engage with the intervention/comparator as intended without assessing receipt or enactment, increases the risk of overlooking important variables along the hypothesised causal pathway to achieving intervention effect. In addition to a sole focus on delivery, when intervention fidelity *is* addressed often only fidelity assessment strategies are focused on, and the use of strategies to enhance or promote intervention fidelity are not always explicitly mentioned (Toomey et al., 2014). This is despite the fact that both types of strategies have distinct and important purposes, as outlined in the introduction (Bellg et al., 2004; Borrelli, 2011; Borrelli et al., 2005). Fidelity enhancement strategies such as treatment manuals or standardisation of provider training play a key role in ensuring the study’s internal validity, and give effective behaviour change interventions the best chance possible to be *proven* effective (Bellg et al., 2004; Horner, Rew, & Torres, 2006).

### Recommendation 2a: Consider fidelity beyond intervention delivery

The existing conceptual debates notwithstanding, intervention fidelity is predominantly viewed as a multi-faceted concept encompassing more than just the delivery of the intervention within the experimental condition. Although suboptimally explored to date, it is also likely that a ‘chain reaction’ relationship exists between domains, e.g. if training is poor, then delivery is likely to be suboptimal; if delivery is poor then intervention receipt and patient understanding is likely to be poor. Therefore, it is important to consider each step of the causal pathway underlying the intended intervention effects and to develop strategies to enhance and assess the fidelity across each of these stages, for both experimental and comparator intervention conditions. The AFFINITIE process evaluation protocol details specifically how fidelity will be enhanced and assessed across each domain of the NIHBCC framework, namely design, training/delivery, receipt and enactment in both intervention and comparator groups. The protocol also details the theoretical underpinnings for the intervention, and a plan for both enhancing and assessing fidelity to these hypothesised causal assumptions, as well as exploring the contextual influences that might influence clinical staffs’ responses to the feedback interventions. By addressing fidelity in such a comprehensive manner, it is clear to see how assumptions are minimised, and how confidence about ‘what happened and why’ during the intervention can be increased.

While not disregarding the importance of comprehensiveness, there are situations where it may not be feasible, necessary or desirable to assess fidelity across all areas. For example, not all fidelity domains may be relevant for all studies, such as the fidelity of provider training for a digital health intervention. Moreover, a study may only have the resources to evaluate one domain, such as the fidelity of intervention delivery by providers. Additionally, specific intervention components may warrant closer investigation and a more in-depth understanding of fidelity than others (Thompson, Lambert, Greaves, & Taylor, 2018). In situations such as this, it is prudent for a study to assess one domain well, rather than all areas poorly. An example is the fidelity assessment conducted by Hardeman et al. within the ProActive trial (Hardeman et al., 2008). The trial evaluated the effectiveness of a complex intervention to increase physical activity among adults at risk of type 2 diabetes (Hardeman et al., 2008); the fidelity assessment focused on one particular and clearly justified uncertainty regarding whether trained facilitators could deliver the complex intervention as planned. In cases such as this, clarity regarding the specific domains of fidelity that are being addressed is crucial, as is a clear and explicit rationale for why specific domains are being targeted. This decision may be based on the existing evidence base, or key uncertainties surrounding the intervention, which may have been highlighted within preceding feasibility studies. Future research could further explore the decision-making processes surrounding how to prioritise or focus fidelity assessments, and also the concept of interdependence between fidelity domains.

### Recommendation 2b: Consider both enhancement and assessment strategies explicitly

As outlined earlier, strategies to enhance and assess fidelity are two distinct elements although there may be inherent overlap between these. For example, self-report checklists could be used to enhance fidelity of delivery by reminding providers of what they are supposed to deliver and to assess fidelity of delivery as part of the evaluation (Bellg et al., 2004). Regardless of potential overlap, it is important to explicitly consider what strategies will be used to enhance intervention fidelity within the trial from the outset, as well as those that will be used to assess it. For example, Carpenter et al. conducted a three-armed trial to compare the effectiveness of paced respiration (intervention) to fast, shallow breathing (attention control) to usual care for the management of hot flashes and other menopausal symptoms (Carpenter, Burns, Wu, Otte, et al., 2013). The strategies used to both enhance and assess intervention fidelity within this trial were explicitly reported in a separate intervention fidelity study (Carpenter, Burns, Wu, Yu, et al., 2013). For example, participants received CDs and DVDs to ensure their understanding of the intervention instructions (i.e. a strategy to enhance treatment receipt). Treatment receipt was assessed during follow-up calls three days after the intervention to verify participants’ ability to understand and follow instructions. This study provides a clear and transparent example of a comprehensive approach to addressing and reporting fidelity in terms of both enhancement and assessment strategies (Carpenter, Burns, Wu, Yu, et al., 2013).

### Issue 3: Limited use of existing fidelity frameworks or guidance

There are currently a number of intervention fidelity frameworks and guidance papers such as the NIHBCC treatment fidelity framework (Borrelli, 2011; Borrelli et al., 2005), the Conceptual Framework of Implementation Fidelity (CFIF) (Carroll et al., 2007), and the Comprehensive Intervention Fidelity Guide (Gearing et al., 2011). They facilitate consideration of fidelity as a broader concept, as well as an explicit focus on strategies to both enhance and assess fidelity from the outset of behaviour change intervention development. However, there is substantial evidence that these frameworks are not being utilised, and that many fidelity assessments fail to reference existing frameworks, models or guidance documents (McGee et al., 2018; Prowse & Nagel, 2015). For example, although the NIHBCC treatment fidelity framework has been in existence for over a decade (Bellg et al., 2004; Borrelli et al., 2005), and was specifically developed for use in studies of health behaviour change interventions, it is often not used in the development or reporting of behavioural trials (Prowse & Nagel, 2015; Robb et al., 2011). In a recent review of measures to assess fidelity of delivery within complex, face-to-face health behaviour change interventions, only 19.7% of the 66 included studies reported using an existing framework to inform the fidelity assessment (Walton et al., 2017). Similarly, a meta-evaluation of fidelity reviews across 10 years of psychosocial research showed that only 23% of included reviews applied a treatment fidelity model or framework in comprehensive detail (Prowse & Nagel, 2015). In relation to trials of behaviour change interventions specifically, 73.6% of participants (*n *= 190) in the aforementioned survey had never used a specific fidelity tool or framework, despite the fact that 68.9% (*n *= 182) had reported previously using strategies to assess and/or enhance fidelity within a trial setting (McGee et al., 2018). Furthermore, where fidelity frameworks are used, they are often modified or adapted without clear or explicit reasons for doing so. For example, previous studies that have applied the NIHBCC Framework used a self-simplified version by summarising framework components (Culloty, Milne, & Sheikh, 2010), or addressed only some of its five domains (Mars et al., 2013; Schober, Sharpe, & Schmidt, 2013) without providing a clear rationale for this, thus limiting the potential to learn from and build on existing literature.

### Recommendation 3: Make use of existing frameworks/guidance

Applying existing guidance or frameworks provides researchers with a more immediate understanding of the potential areas of an intervention where fidelity may need to be both enhanced and assessed, saving valuable time and effort. In addition, building upon existing research in this way will help structure and standardise research, and improve subsequent comparability and ability to synthesise findings. For example, Toomey et al. (2016) provides an example of the development of a protocol to enhance and assess intervention fidelity within a complex self-management behaviour change intervention for people with chronic pain, using the NIHBCC fidelity framework, and how each component of the framework will be addressed or not in the subsequent trial. Where adaptations or omissions are made to existing frameworks (e.g. removal of specific components or elements), this should be made explicit and reasons for doing so provided, as detailed by Hasson (2010) in the modified CFIF (Hasson, 2010). In this way, we can generate a better understanding of the gaps in the evidence base, what the potential inadequacies of current frameworks are, and where future refinements could be made to ensure that they are fit for purpose.

The reasons why existing frameworks are not fully utilised have not been investigated to date. However, the McGee et al. survey identified a clear need for more practical guidance (McGee et al., 2018). It may be that existing frameworks do not sufficiently consider the pragmatics of addressing intervention fidelity across the spectrum of intervention research, e.g. from feasibility to effectiveness studies to real-world implementation. As such, future fidelity research could conduct more comparative work on existing frameworks, i.e. investigating what frameworks exist, similarities and differences of these frameworks, and contexts or intervention stages for which frameworks may be most appropriate. A systematic scoping review is currently underway into existing conceptual fidelity frameworks and models (Roy et al., 2018). This review will facilitate such an investigation as well as the application and uptake of existing frameworks.

### Issue 4: Lack of focus on quality and comprehensiveness of fidelity assessment strategies

The quality and comprehensiveness of methods used for assessing fidelity are of great importance to ensure reliable and valid information. Although a combination of quantitative and qualitative methods has been previously suggested to comprehensively assess intervention fidelity (Bellg et al., 2004; Mowbray et al., 2003; Poltawski, Norris, & Dean, 2014; Spillane et al., 2007; Toomey & Hardeman, 2017), intervention fidelity is often only assessed using quantitative methods (French et al., 2015; Lorencatto, West, Christopherson, & Michie, 2013; Walton et al., 2017). This is problematic as the use of quantitative methods in isolation to assess intervention fidelity may not allow for exploration of variation in findings or the factors influencing fidelity (Lorencatto et al., 2013).

In addition, recent reviews of fidelity in trials of behaviour change interventions have explored and examined the quality and properties of intervention fidelity assessment measures (Lambert et al., 2017; Prowse & Nagel, 2015; Rixon et al., 2016; Walton et al., 2017). Rixon et al. found that 78.2% of studies that assessed treatment receipt conducted self-report assessments only, and only 26% reported on the reliability or validity of measures used (Rixon et al., 2016). Another review by Walton et al. showed that 74.2% of included studies reported on either the reliability or validity of pre-existing and/or self-developed fidelity measures used to assess treatment delivery or participant engagement (Walton et al., 2017). However, this review also found that both the reliability and validity of a measure were only reported in one study. Moreover, only 25.8% of included studies reported the implementation properties of measures used such as cost, acceptability and practicality (Walton et al., 2017). Practical issues such as time and cost restraints are a common concern in developing appropriate strategies to enhance and assess intervention fidelity (Breitenstein et al., 2010; Perepletchikova, Hilt, Chereji, & Kazdin, 2009), and are among the most significant barriers to addressing intervention fidelity within trials of health behaviour change interventions (McGee et al., 2018). However, the current evidence suggests that these important properties of fidelity measures have been overlooked and under-addressed in existing trials. This limits the potential to inform the selection of assessment strategies by other researchers, and the contribution that these fidelity assessments can make towards the interpretation of the trial findings.

### Recommendation 4: Consider the psychometric and implementation properties of mixed method fidelity assessment strategies

The development and use of fidelity assessment strategies with good psychometric properties are crucial to advance the evidence base. Existing validated and reliable fidelity measures should be considered where possible, e.g. the Motivational Interviewing Treatment Integrity Code (Moyers, Rowell, Manuel, Ernst, & Houck, 2016); however, it is acknowledged that these do not always exist, nor are they always feasible to develop, or even desirable (Walton et al., 2017). The paper by Dima (2018) in this Special Issue provides a step-by-step approach to determining core psychometric properties for self-developed measures that may be of use in guiding development and/or evaluation of fidelity measures (Dima, 2018). We also recommend the appropriate use of mixed methods to combine the strengths of both qualitative and quantitative approaches and generate accurate and more comprehensive fidelity findings (Shaw, Hiles, West, Holland, & Gwyther, 2018). For example, using quantitative data collection, Toomey, Matthews, and Hurley (2017) found that physiotherapists who were longer qualified delivered an education and exercise intervention with less fidelity; by integrating these findings with semi-structured interviews with the physiotherapists, they identified the reasons for this to be spending too much time on familiar intervention components at the expense of other components. In a similar way, Williams et al. (2020) integrated quantitative fidelity assessments of the delivery of a walking intervention by nurses and healthcare assistants with qualitative patient interviews regarding treatment receipt; thus facilitating a broader understanding of patient, provider and component-level factors influencing intervention fidelity across multiple domains (Williams et al., 2020).

Notwithstanding the importance of obtaining a comprehensive understanding of fidelity, implementation properties (i.e. cost, feasibility and practicality) are also of the utmost importance. As such, selection of fidelity assessment strategies (whether quantitative, qualitative or mixed methods) often means balancing comprehensiveness with feasibility (Toomey & Hardeman, 2017). Given the realities of resource restraints within trials of behavioural interventions, we recommend that fidelity enhancement and assessment strategies are properly costed for during grant applications, rather than as an afterthought. To facilitate the development of feasible and comprehensive mixed methods assessment strategies, future research could explore the concept of research efficiency in fidelity i.e. how to select and analyse sufficient amounts of fidelity data without sacrificing information provided, and how best to embed fidelity assessments within routine trial data collection. In addition, further research could also explore the potential for generic valid and reliable fidelity assessment instruments, as has been done recently with the development of three generic measures for intervention acceptability, appropriateness and feasibility (Weiner et al., 2017).

### Issue 5: Lack of explicit focus on the balance between fidelity and adaptation

The need for balancing intervention fidelity with adaptation is a recurring theme within the broader intervention fidelity body of literature. Previous research has debated the concept of fidelity versus adaptation, with the case made for both strict intervention fidelity and for allowing and encouraging adaptation (Dane & Schneider, 1998; Dusenbury et al., 2003). For many, a rigid focus on intervention fidelity is seen to restrict therapeutic freedom, viewed as detrimental to intervention outcomes (Greenhalgh & Papoutsi, 2018). This concern regarding a lack of flexibility has been previously identified as a barrier to addressing intervention fidelity both within health behaviour change and psychotherapy interventions (McGee et al., 2018; Perepletchikova et al., 2009). However, others view a tighter focus on fidelity as essential to ensure greater validity of the intervention evaluation, as well as a more transparent intervention process (Hogue et al., 2008; Holliday, Audrey, Moore, Parry-Langdon, & Campbell, 2009; Moskowitz, 1993). To date, evidence is inconclusive regarding whether adaptations have a positive, negative or neutral effect on intervention outcomes (Breitenstein et al., 2010). With this in mind, more recently, it is suggested that achieving an appropriate balance between fidelity and adaptation is needed and that in certain circumstances adaptation may in fact be a critical element of a behaviour change intervention (Hawe, Shiell, & Riley, 2004; Holliday et al., 2009; Kirk et al., 2019; McHugh et al., 2009). However, despite the clear need for careful and explicit consideration of intervention adaptation and what is considered ‘appropriate’ or desirable, several systematic reviews have shown that this particular aspect remains an under-addressed area of health behaviour change intervention research (Durlak & DuPre, 2008; Yamato, Maher, Saragiotto, Hoffmann, & Moseley, 2016; Yu, Balasubramanaiam, Offringa, & Kelly, 2018).

### Recommendation 5: Consider the need for balance between fidelity and adaptation a-priori

In the absence of clear consensus regarding the best ways of addressing adaptation, we recommend that researchers view the issue of fidelity and adaptation not as an ‘either or’, but as two sides of the same coin. As such, researchers should consider the issue of adaptation during the stages of intervention development, alongside the development of plans for enhancing, assessing and reporting intervention fidelity, rather than at the end of the trial. Central to this should be careful consideration and explication of what the core components of the intervention are, and where possible, to consider from the outset what intervention adaptations may be acceptable or not (Holliday et al., 2009). For example, several researchers have posited the concept of theoretical versus content fidelity. Depending on the context and research questions, it may be more appropriate and indeed important to enable the specific ‘form’ an intervention component takes (e.g. a video or booklet), or the operationalisation of a behaviour change technique (BCT) to be adapted, as long as it retains fidelity to the underlying intervention theory (Keogh, Matthews, & Hurley, 2018) or the ‘function’ of the component or BCT (e.g. to ‘provide instruction on how to perform the behaviour’) (Hawe, Shiell, & Riley, 2004; Hawe, Shiell, Riley, & Gold, 2004; Kirk et al., 2019; McHugh et al., 2009). Clear specification of the intervention components and hypothesised mechanisms of change using logic models and taxonomies such as the BCT Taxonomy v1 (Michie et al., 2013) is therefore essential to facilitate this. Achieving a balance between fidelity and adaptation may also vary depending on the stage of the intervention (Araújo-Soares, Hankonen, Presseau, Rodrigues, & Sniehotta, 2018; Castro, Barrera, & Martinez, 2004; Dumas, Lynch, Laughlin, Phillips Smith, & Prinz, 2001; Durlak & DuPre, 2008; Hasson, Blomberg, & Duner, 2012; Holliday et al., 2009). For example, within feasibility studies the focus may need to be on exploring how and why adaptations occur to determine which are avoidable and/or unacceptable (Araújo-Soares et al., 2018; Holliday et al., 2009); within an effectiveness trial, the focus may need to be on ensuring high fidelity to core components, permitting only ‘acceptable’ adaptations (Dumas et al., 2001; Holliday et al., 2009; McHugh et al., 2009), and this may differ entirely again for implementation studies. Regardless of how this balance is determined, an appropriate evaluation of intervention fidelity and adaptation is key. There is much research to be done in this area, one of which could be to clarify more thoroughly the relative role of adaptation across intervention stages. Moreover, current approaches to optimising behavioural intervention design and evaluation by building in adaptations, such as Multiphase Optimization Strategy (MOST) and Sequential Multiple Assignment Randomized Trial (SMART) (Collins, Murphy, & Strecher, 2007), offer further opportunities to explore the concepts of fidelity and adaptation.

### Issue 6: Poor reporting of how intervention fidelity is addressed

Several systematic reviews have highlighted that the capacity to examine fidelity within included studies is potentially limited by poor reporting in published studies (McArthur et al., 2012; Toomey et al., 2014; Toomey et al., 2018). Within the survey of researchers and healthcare professionals with trial experience by McGee et al., 13.8% of participants described always using strategies to enhance and assess fidelity of behavioural interventions within a trial, while only 5.5% described always *reporting* the use of these strategies (McGee et al., 2018). Moreover, when information on intervention fidelity *is* reported, it often focuses on how fidelity was assessed, but rarely reports who conducted the assessment or what the findings were (McGee et al., 2018; Toomey et al., 2018). This is problematic as there are both pros and cons in terms of whether fidelity is assessed independently, or by a blinded/unblinded trial study member, as outlined within the MRC process evaluation guidance (Moore et al., 2015). This also limits the potential for the use of fidelity data to interpret trial findings, defeating the purpose of conducting the fidelity assessment in the first instance. Adequate reporting of how intervention fidelity has been enhanced and assessed is of paramount importance for progressing the science in this field. Although several reporting criteria relevant to trials of health behaviour change interventions exist, such as the Transparent Reporting of Evaluations with Nonrandomized Designs (TREND) (Des Jarlais, Lyles, & Crepaz, 2004), Consolidated Standards of Reporting Trials (CONSORT) (Schulz, Altman, & Moher, 2010) and the Template for Intervention Description and Replication (TIDieR) (Hoffmann et al., 2014) criteria, fidelity is not alluded to at all in the CONSORT statement, and only briefly mentioned within TREND and the recent CONSORT-SPI extension (Montgomery et al., 2018). Although the TIDieR checklist contains four items (items 9–12) relevant to the reporting of fidelity and adaptation, other important aspects of fidelity as highlighted in this manuscript (e.g. quality of fidelity assessment strategies, domains other than delivery) are not addressed in any of these guidelines.

### Recommendation 6: Comprehensively report use of strategies to enhance and assess fidelity and results of fidelity assessments

To enhance transparency, comparability and replication, we recommend that researchers report any fidelity frameworks used, strategies used to enhance or assess fidelity, their methodological properties and who has conducted them, as well as fidelity assessment findings and any adaptations across all fidelity domains. We also recommend that authors report how the findings of fidelity assessments are conducted (e.g. to prevent intervention drift over time), and when they are conducted in relation to the main trial results, for example, without knowledge of trial findings, or subsequently integrated to illuminate the findings. Bova et al. (2017) provide an example of how fidelity of intervention delivery was monitored over time, and data were subsequently used to correct drift in both the intervention and comparator conditions during the course of a complex clinical trial (Bova et al., 2017). They also explicitly report using fidelity of delivery data within their main trial analysis to examine the effect of fidelity variability on treatment outcomes (Sullivan-Bolyai et al., 2015). Despite some good examples of the use of fidelity data for interpreting trial findings, clear guidance on how to do this is lacking. Further research could explore how and when to best integrate fidelity data with trial outcomes, especially if considering fidelity data from more than one domain (e.g. fidelity of training or receipt), or mixed methods fidelity findings. In addition, future work could explore the development of new or the refinement of existing reporting criteria, such as the TIDieR checklist (Hoffmann et al., 2014), to enhance how fidelity is reported across all domains. Journal editors and reviewers could also facilitate and encourage the reporting of this information within supplementary material or elsewhere.

## Conclusion

Intervention fidelity is an area of key methodological importance which needs to be improved in order to enhance the science of behaviour change. The aforementioned issues are not intended to be an exhaustive list, rather to create awareness of common issues of particular importance for improving intervention fidelity within trials of health behaviour change interventions. This article outlined specific issues concerning intervention fidelity within trials of health behaviour change interventions and provides practical recommendations to be considered by researchers in order to improve how fidelity is addressed (summarised in Table 2). In addition to the issues and recommendations presented here, the article highlights areas for future research to better understand the challenges in addressing intervention fidelity and develop potential solutions. Areas for future research include the examination of existing fidelity frameworks and reporting guidance, exploration of research efficiency in fidelity and the balance between fidelity and adaptation, and guidance for how to best integrate fidelity data with trial outcomes.

**Table 2.** Practical recommendations for addressing key methodological intervention fidelity issues.

Overarching issue	Specific recommendations
1. Lack of standardisation regarding how fidelity is conceptualised and defined	1. Clarify how fidelity is defined and conceptualised
2. Limited focus beyond assessing of fidelity of delivery	2a. Consider fidelity beyond intervention delivery 2b. Consider both enhancement and assessment strategies explicitly
3. Limited use of existing fidelity frameworks or guidance	3. Make use of existing frameworks
4. Lack of focus on quality and comprehensiveness of fidelity assessment strategies	4. Consider the psychometric and implementation properties of mixed method fidelity assessment strategies
5. Lack of explicit focus on the balance between fidelity and adaptation	5. Consider the need for balance between fidelity and adaptation a-priori
6. Poor reporting of how intervention fidelity is addressed	6. Comprehensively report use of strategies to enhance and assess fidelity and results of fidelity assessments

## References

[CIT0001] Abry, T., Hulleman, C. S., & Rimm-Kaufman, S. E. (2014). Using indices of fidelity to intervention core components to identify program active ingredients. *American Journal of Evaluation*, *36*, 320–338. doi: 10.1177/1098214014557009

[CIT0002] Araújo-Soares, V., Hankonen, N., Presseau, J., Rodrigues, A., & Sniehotta, F. F. (2018). Developing behavior change interventions for self-management in chronic illness. *European Psychologist*, *24*(1), 7–25. 10.1027/1016-9040/a000330 31496632 PMC6727632

[CIT0003] Bacon, S. L., Lavoie, K. L., Ninot, G., Czajkowski, S., Freedland, K. E., Michie, S., … Spring, B. (2014). An international perspective on improving the quality and potential of behavioral clinical trials. *Current Cardiovascular Risk Reports*, *9*, 427. doi: 10.1007/s12170-014-0427-0

[CIT0004] Baer, J. S., Ball, S. A., Campbell, B. K., Miele, G. M., Schoener, E. P., & Tracy, K. (2007). Training and fidelity monitoring of behavioral interventions in multi-site addictions research. *Drug and Alcohol Dependence*, *87*, 107–118. doi: 10.1016/j.drugalcdep.2006.08.028 17023123 PMC1876726

[CIT0005] Banerjee, A., Chitnis, U. B., Jadhav, S. L., Bhawalkar, J. S., & Chaudhury, S. (2009). Hypothesis testing, type I and type II errors. *Industrial Psychiatry Journal*, *18*, 127–131. doi: 10.4103/0972-6748.62274 21180491 PMC2996198

[CIT0006] Bellg, A. J., Borrelli, B., Resnick, B., Hecht, J., Minicucci, D. S., Ory, M., … Czajkowski, S. (2004). Enhancing treatment fidelity in health behavior change studies: Best practices and recommendations from the NIH Behavior Change Consortium. *Health Psychology*, *23*, 443–451. doi: 10.1037/0278-6133.23.5.443 15367063

[CIT0007] Borrelli, B. (2011). The assessment, monitoring, and enhancement of treatment fidelity in public health clinical trials. *Journal of Public Health Dentistry*, *71*, S52–S63. doi: 10.1111/j.1752-7325.2011.00233.x PMC3074245 21499543

[CIT0008] Borrelli, B., Sepinwall, D., Ernst, D., Bellg, A. J., Czajkowski, S., Breger, R., … Orwig, D. (2005). A new tool to assess treatment fidelity and evaluation of treatment fidelity across 10 years of health behavior research. *Journal of Consulting and Clinical Psychology*, *73*, 852–860. doi: 10.1037/0022-006X.73.5.852 16287385

[CIT0009] Bova, C., Jaffarian, C., Crawford, S., Quintos, J. B., Lee, M., & Sullivan-Bolyai, S. (2017). Intervention fidelity: monitoring drift, providing feedback, and assessing the control condition. *Nursing Research*, *66*, 54–59. doi: 10.1097/NNR.0000000000000194 27977568 PMC5172380

[CIT0010] Breitenstein, S. M., Gross, D., Garvey, C. A., Hill, C., Fogg, L., & Resnick, B. (2010). Implementation fidelity in community-based interventions. *Research in Nursing & Health*, *33*, 164–173. doi: 10.1002/nur.20373 20198637 PMC3409469

[CIT0011] Carpenter, J. S., Burns, D. S., Wu, J., Otte, J. L., Schneider, B., Ryker, K., … Yu, M. (2013). Paced respiration for vasomotor and other menopausal symptoms: A randomized, controlled trial. *Journal of General Internal Medicine*, *28*, 193–200. doi: 10.1007/s11606-012-2202-6 22936289 PMC3614127

[CIT0012] Carpenter, J. S., Burns, D. S., Wu, J., Yu, M., Ryker, K., Tallman, E., & Von Ah, D. (2013). Strategies used and data obtained during treatment fidelity monitoring. *Nursing Research*, *62*, 59–65. doi: 10.1097/NNR.0b013e31827614fd 23222844 PMC3523275

[CIT0013] Carroll, C., Patterson, M., Wood, S., Booth, A., Rick, J., & Balain, S. (2007). A conceptual framework for implementation fidelity. *Implementation Science*, *2*, 40. doi: 10.1186/1748-5908-2-40 18053122 PMC2213686

[CIT0014] Castro, F., Barrera, M., & Martinez, C. (2004). The cultural adaptation of prevention interventions: Resolving tensions between fidelity and fit. *Prevention Science*, *5*, 41–45. doi: 10.1023/B:PREV.0000013980.12412.cd 15058911

[CIT0015] Collins, L. M., Murphy, S. A., & Strecher, V. (2007). The multiphase optimization strategy (MOST) and the sequential multiple assignment randomized trial (SMART): New methods for more potent ehealth interventions. *American Journal of Preventive Medicine*, *32*, S112–S118. doi: 10.1016/j.amepre.2007.01.022 17466815 PMC2062525

[CIT0016] Craig, P., Dieppe, P., Macintyre, S., Michie, S., Nazareth, I., & Petticrew, M. (2008). Developing and evaluating complex interventions: The new Medical Research Council guidance. *BMJ*, *337*, A1655. doi: 10.1136/bmj.a1655 18824488 PMC2769032

[CIT0017] Culloty, T., Milne, D. L., & Sheikh, A. I. (2010). Evaluating the training of clinical supervisors: A pilot study using the fidelity framework. *The Cognitive Behaviour Therapist*, *3*, 132–144. doi: 10.1017/S1754470X10000139

[CIT0018] Dane, A., & Schneider, B. (1998). Program integrity in primary and early secondary prevention: Are implementation effects out of control? *Clinical Psychology Review*, *18*, 23–45. doi: 10.1016/S0272-7358(97)00043-3 9455622

[CIT0019] De Bruin, M., Black, N., Javornik, N., Viechtbauer, W., Eisma, M. C., Hartman-Boyce, J., … Johnston, M. (2020). Underreporting of the active content of behavioural interventions: A systematic review and meta-analysis of randomised trials of smoking cessation interventions. *Health Psychology Review*. 10.1080/17437199.2019.1709098 31906781

[CIT0020] Des Jarlais, D., Lyles, C., & Crepaz, N. (2004). Improving the reporting quality of nonrandomized evaluations of behavioral and public health interventions: The trend statement. *American Journal of Public Health*, *94*, 361–366. doi: 10.2105/AJPH.94.3.361 14998794 PMC1448256

[CIT0021] Dima, A. L. (2018). Scale validation in applied health research: Tutorial for a 6-step R-based psychometrics protocol. *Health Psychology And Behavioral Medicine*, *6*, 136–161. doi: 10.1080/21642850.2018.1472602 PMC8133536 34040826

[CIT0022] Di Rezze, B. (2012). *Ensuring intervention fidelity in rehabilitation research*. CanChild Centre for Childhood Disability Research.

[CIT0023] Dumas, J. E., Lynch, A. M., Laughlin, J. E., Phillips Smith, E., & Prinz, R. J. (2001). Promoting intervention fidelity. Conceptual issues, methods, and preliminary results from the early alliance prevention trial. *American Journal of Preventive Medicine*, *20*, 38–47. doi: 10.1016/S0749-3797(00)00272-5 11146259

[CIT0024] Durlak, J. A., & DuPre, E. P. (2008). Implementation matters: A review of research on the influence of implementation on program outcomes and the factors affecting implementation. *American Journal of Community Psychology*, *41*, 327–350. doi: 10.1007/s10464-008-9165-0 18322790

[CIT0025] Dusenbury, L., Brannigan, R., Falco, M., & Hansen, W. (2003). A review of research on fidelity of implementation: Implications for drug abuse prevention in school settings. *Health Education Research*, *18*, 237–256. doi: 10.1093/her/18.2.237 12729182

[CIT0026] Feldman, M. B., Silapaswan, A., Schaefer, N., & Schermele, D. (2014). Is there life after Debi? Examining health behavior maintenance in the diffusion of effective behavioral interventions initiative. *American Journal of Community Psychology*, *53*, 286–313. doi: 10.1007/s10464-014-9629-3 24499926

[CIT0027] Flodgren, G., Gonçalves-Bradley, D. C., & Summerbell, C. D. (2017). Interventions to change the behaviour of health professionals and the organisation of care to promote weight reduction in children and adults with overweight or obesity. *Cochrane Database of Systematic Reviews*, (11), Art. No.: CD000984. 10.1002/14651858.CD000984.pub3 PMC6486102 29190418

[CIT0028] French, S. D., Green, S. E., Francis, J. J., Buchbinder, R., O'connor, D. A., Grimshaw, J. M., & Michie, S. (2015). Evaluation of the fidelity of an interactive face-to-face educational intervention to improve general practitioner management of back pain. *BMJ Open*, *5*, e007886. 10.1136/bmjopen-2015-007886 PMC4499726 26155819

[CIT0029] Garbacz, L., Brown, D., Spee, G., Polo, A., & Budd, K. (2014). Establishing treatment fidelity in evidence-based parent training programs for externalizing disorders in children and adolescents. *Clinical Child and Family Psychology Review*, *17*, 230–247. doi: 10.1007/s10567-014-0166-2 24706293

[CIT0030] Gearing, R. E., El-Bassel, N., Ghesquiere, A., Baldwin, S., Gillies, J., & Ngeow, E. (2011). Major ingredients of fidelity: A review and scientific guide to improving quality of intervention research implementation. *Clinical Psychology Review*, *31*, 79–88. doi: 10.1016/j.cpr.2010.09.007 21130938

[CIT0031] Greenhalgh, T., & Papoutsi, C. (2018). Studying complexity in health services research: Desperately seeking an overdue paradigm shift. *BMC Medicine*, *16*, 95. doi: 10.1186/s12916-018-1089-4 29921272 PMC6009054

[CIT0032] Hankonen, N., Heino, M. T. J., Hynynen, S.-T., Laine, H., Araújo-Soares, V., Sniehotta, F. F., … Haukkala, A. (2017). Randomised controlled feasibility study of a school-based multi-level intervention to increase physical activity and decrease sedentary behaviour among vocational school students. *International Journal of Behavioral Nutrition and Physical Activity*, *14*, 37. doi: 10.1186/s12966-017-0484-0 PMC5361824 28327174

[CIT0033] Hankonen, N., Sutton, S., Prevost, A. T., Simmons, R. K., Griffin, S. J., Kinmonth, A. L., & Hardeman, W. (2015). Which behavior change techniques are associated with changes in physical activity, diet and body mass index in people with recently diagnosed diabetes? *Annals of Behavioral Medicine*, *49*, 7–17. doi: 10.1007/s12160-014-9624-9 24806469 PMC4335098

[CIT0034] Hardeman, W., Michie, S., Fanshawe, T., Prevost, A. T., Mcloughlin, K., & Kinmonth, A. L. (2008). Fidelity of delivery of a physical activity intervention: Predictors and consequences. *Psychology Health*, *23*, 11–24. doi: 10.1080/08870440701615948 25159904

[CIT0035] Hasson, H. (2010). Systematic evaluation of implementation fidelity of complex interventions in health and social care. *Implementation Science*, *5*, 67. doi: 10.1186/1748-5908-5-67 20815872 PMC2942793

[CIT0036] Hasson, H., Blomberg, S., & Duner, A. (2012). Fidelity and moderating factors in complex interventions: A case study of a continuum of care program for frail elderly people in health and social care. *Implementation Science*, *7*, 23. doi: 10.1186/1748-5908-7-23 22436121 PMC3342887

[CIT0037] Hawe, P., Shiell, A., & Riley, T. (2004). Complex interventions: How “out of control” can a randomised controlled trial be? *BMJ*, *328*, 1561. doi: 10.1136/bmj.328.7455.1561 15217878 PMC437159

[CIT0038] Hawe, P., Shiell, A., Riley, T., & Gold, L. (2004). Methods for exploring implementation variation and local context within a cluster randomised community intervention trial. *Journal of Epidemiology Community Health*, *58*, 788–793. doi: 10.1136/jech.2003.014415 15310806 PMC1732876

[CIT0039] Hoffmann, T. C., Glasziou, P. P., Boutron, I., Milne, R., Perera, R., Moher, D., … Michie, S. (2014). Better reporting of interventions: Template for intervention description and replication (TIDieR) checklist and guide. *BMJ*, *348*, 1–12. 10.1136/bmj.g1687 24609605

[CIT0040] Hogue, A., Henderson, C. E., Dauber, S., Barajas, P. C., Fried, A., & Liddle, H. A. (2008). Treatment adherence, competence, and outcome in individual and family therapy for adolescent behavior problems. *Journal of Consulting and Clinical Psychology*, *76*, 544–555. doi: 10.1037/0022-006X.76.4.544 18665684 PMC2843085

[CIT0041] Holliday, J., Audrey, S., Moore, L., Parry-Langdon, N., & Campbell, R. (2009). High fidelity? How should we consider variations in the delivery of school-based health promotion interventions? *Health Education Journal*, *68*, 44–62. doi: 10.1177/0017896908100448

[CIT0042] Horner, S., Rew, L., & Torres, R. (2006). Enhancing intervention fidelity: A means of strengthening study impact. *Journal for Specialists in Pediatric Nursing*, *11*, 80–89. doi: 10.1111/j.1744-6155.2006.00050.x 16635187 PMC1474027

[CIT0043] JaKa, M. M., Haapala, J. L., Trapl, E. S., Kunin-Batson, A. S., Olson-Bullis, B. A., Heerman, W. J., … Sherwood, N. E. (2016). Reporting of treatment fidelity in behavioural paediatric obesity intervention trials: A systematic review. *Obesity Reviews*, *17*, 1287–1300. doi: 10.1111/obr.12464 27612933 PMC5193220

[CIT0044] Jepson, R. G., Harris, F. M., Platt, S., & Tannahill, C. (2010). The effectiveness of interventions to change six health behaviours: A review of reviews. *BMC Public Health*, *10*, 538. doi: 10.1186/1471-2458-10-538 20825660 PMC2944371

[CIT0045] Johnson-Kozlow, M., Hovell, M. F., Rovniak, L. S., Sirikulvadhana, L., Wahlgren, D. R., & Zakarian, J. M. (2008). Fidelity issues in secondhand smoking interventions for children. *Nicotine Tobacco Research*, *10*, 1677–1690. doi: 10.1080/14622200802443429 19023822 PMC3533496

[CIT0046] Keogh, A., Matthews, J., & Hurley, D. A. (2018). An assessment of physiotherapist’s delivery of behaviour change techniques within the SOLAS feasibility trial. *British Journal of Health Psychology*, *23*(4), 908–932. 10.1111/bjhp.12323 29888520

[CIT0047] Kirk, M. A., Haines, E. R., Rokoske, F. S., Powell, B. J., Weinberger, M., Hanson, L. C., & Birken, S. A. (2019). A case study of a theory-based method for identifying and reporting core functions and forms of evidence-based interventions. *Translational Behavioral Medicine*, *ibz178*. 10.1093/tbm/ibz178 PMC7877297 31793635

[CIT0048] Köykkä, K., Absetz, P., Araújo-Soares, V., Knittle, K., Sniehotta, F. F., & Hankonen, N. (2019). Combining the reasoned action approach and habit formation to reduce sitting time in classrooms: Outcome and process evaluation of the let’s move it teacher intervention. *Journal of Experimental Social Psychology*, *81*, 27–38. doi: 10.1016/j.jesp.2018.08.004

[CIT0049] Kwasnicka, D., Dombrowski, S. U., White, M., & Sniehotta, F. (2016). Theoretical explanations for maintenance of behaviour change: A systematic review of behaviour theories. *Health Psychology Review*, *10*, 277–296. doi: 10.1080/17437199.2016.1151372 26854092 PMC4975085

[CIT0050] Lambert, J. D., Greaves, C. J., Farrand, P., Cross, R., Haase, A. M., & Taylor, A. H. (2017). Assessment of fidelity in individual level behaviour change interventions promoting physical activity among adults: A systematic review. *BMC Public Health*, *17*, 765. doi: 10.1186/s12889-017-4778-6 28969669 PMC5625828

[CIT0051] Lichstein, K. L., Riedel, B. W., & Grieve, R. (1994). Fair tests of clinical trials: A treatment implementation model. *Advances in Behaviour Research and Therapy*, *16*, 1–29. doi: 10.1016/0146-6402(94)90001-9

[CIT0052] Lorencatto, F., Gould, N. J., Mcintyre, S. A., During, C., Bird, J., Walwyn, R., … Francis, J. J. (2016). A multidimensional approach to assessing intervention fidelity in a process evaluation of audit and feedback interventions to reduce unnecessary blood transfusions: A study protocol. *Implementation Science*, *11*, 163. doi: 10.1186/s13012-016-0528-x 27955683 PMC5153878

[CIT0053] Lorencatto, F., West, R., Christopherson, C., & Michie, S. (2013). Assessing fidelity of delivery of smoking cessation behavioural support in practice. *Implementation Science*, *8*, 40. doi: 10.1186/1748-5908-8-40 23557119 PMC3622616

[CIT0054] Mars, T., Ellard, D., Carnes, D., Homer, K., Underwood, M., & Taylor, S. J. C. (2013). Fidelity in complex behaviour change interventions: A standardised approach to evaluate intervention integrity. *BMJ Open*, *3*(11), e003555. 10.1136/bmjopen-2013-003555 PMC3831105 24240140

[CIT0055] McArthur, B. A., Riosa, P. B., & Preyde, M. (2012). Treatment fidelity in psychosocial intervention for children and adolescents with comorbid problems. *Child And Adolescent Mental Health*, *17*, 139–145. doi: 10.1111/j.1475-3588.2011.00635.x 32847270

[CIT0056] McCrabb, S., Lane, C., Hall, A., Milat, A., Bauman, A., Sutherland, R., … Wolfenden, L. (2019). Scaling-up evidence-based obesity interventions: A systematic review assessing intervention adaptations and effectiveness and quantifying the scale-up penalty. *Obesity Reviews*, *20*(7), 964–982. 10.1111/obr.12845 30868745

[CIT0057] McGee, D., Lorencatto, F., Matvienko-Sikar, K., & Toomey, E. (2018). Surveying knowledge, practice and attitudes towards intervention fidelity within trials of complex healthcare interventions. *Trials*, *19*, 504. doi: 10.1186/s13063-018-2838-6 30231917 PMC6147031

[CIT0058] McHugh, R. K., Murray, H. W., & Barlow, D. H. (2009). Balancing fidelity and adaptation in the dissemination of empirically-supported treatments: The promise of transdiagnostic interventions. *Behaviour Research and Therapy*, *47*, 946–953. doi: 10.1016/j.brat.2009.07.005 19643395 PMC2784019

[CIT0059] Michie, S., Richardson, M., Johnston, M., Abraham, C., Francis, J., Hardeman, W., … Wood, C. E. (2013). The behavior change technique taxonomy (V1) of 93 hierarchically clustered techniques: Building an international consensus for the reporting of behavior change interventions. *Annals of Behavioral Medicine*, *46*(1), 81–95. 10.1007/s12160-013-9486-6 23512568

[CIT0060] Moncher, F. J., & Prinz, R. J. (1991). Treatment fidelity in outcome studies. *Clinical Psychology Review*, *11*, 247–266. doi: 10.1016/0272-7358(91)90103-2

[CIT0061] Montgomery, P., Grant, S., Mayo-Wilson, E., Macdonald, G., Michie, S., Hopewell, S., & Moher, D. (2018). Reporting randomised trials of social and psychological interventions: The CONSORT-SPI 2018 extension. *Trials*, *19*, 407. doi: 10.1186/s13063-018-2733-1 30060754 PMC6066921

[CIT0062] Montgomery, P., Underhill, K., Gardner, F., Operario, D., & Mayo-Wilson, E. (2013). The Oxford implementation index: A new tool for incorporating implementation data into systematic reviews and meta-analyses. *Journal of Clinical Epidemiology*, *66*, 874–882. doi: 10.1016/j.jclinepi.2013.03.006 23810026 PMC3746185

[CIT0063] Moore, G. F., Audrey, S., Barker, M., Bond, L., Bonell, C., Hardeman, W., … Baird, J. (2015). Process evaluation of complex interventions: Medical research council guidance. *BMJ*, *350*(h1258). 10.1136/bmj.h1258 PMC4366184 25791983

[CIT0064] Moskowitz, J. M. (1993). Why reports of outcome evaluations are often biased or uninterpretable: Examples from evaluations of drug abuse prevention programs. *Evaluation and Program Planning*, *16*, 1–9. doi: 10.1016/0149-7189(93)90032-4

[CIT0065] Mowbray, C. T., Holter, M. C., Teague, G. B., & Bybee, D. (2003). Fidelity criteria: Development, measurement, and validation. *American Journal of Evaluation*, *24*, 315–340. doi: 10.1177/109821400302400303

[CIT0066] Moyers, T. B., Rowell, L. N., Manuel, J. K., Ernst, D., & Houck, J. M. (2016). The motivational interviewing treatment integrity code (MITI 4): Rationale, preliminary reliability and validity. *Journal of Substance Abuse Treatment*, *65*, 36–42. doi: 10.1016/j.jsat.2016.01.001 26874558 PMC5539964

[CIT0067] O’Shea, O., Mccormick, R., Bradley, J. M., & O’Neill, B. (2016). Fidelity review: A scoping review of the methods used to evaluate treatment fidelity in behavioural change interventions. *Physical Therapy Reviews*, *21*, 207–214. doi: 10.1080/10833196.2016.1261237

[CIT0068] Parham, L. D., Cohn, E. S., Spitzer, S., Koomar, J. A., Miller, L. J., Burke, J. P., … Summers, C. A. (2007). Fidelity in sensory integration intervention research. *American Journal of Occupational Therapy*, *61*, 216–227. doi: 10.5014/ajot.61.2.216 17436844

[CIT0069] Perepletchikova, F., Hilt, L. M., Chereji, E., & Kazdin, A. E. (2009). Barriers to implementing treatment integrity procedures: Survey of treatment outcome researchers. *Journal of Consulting and Clinical Psychology*, *77*, 212–218. doi: 10.1037/a0015232 19309181

[CIT0070] Poltawski, L., Norris, M., & Dean, S. (2014). Intervention fidelity: Developing an experience-based model for rehabilitation research. *Journal of Rehabilitation Medicine*, *46*, 609–615. doi: 10.2340/16501977-1848 24940792

[CIT0071] Prowse, P. T., & Nagel, T. (2015). A meta-evaluation: The role of treatment fidelity within psychosocial interventions during the last decade. *Journal of Psychiatry*, *18*, 1–7.

[CIT0072] Quay, H. C. (1977). The three faces of evaluation: What can be expected to work. *Correctional Psychologist*, *4*, 341–354. doi: 10.1177/009385487700400402

[CIT0073] Rixon, L., Baron, J., Mcgale, N., Lorencatto, F., Francis, J., & Davies, A. (2016). Methods used to address fidelity of receipt in health intervention research: A citation analysis and systematic review. *BMC Health Services Research*, *16*, 663. doi: 10.1186/s12913-016-1904-6 27863484 PMC5116196

[CIT0074] Robb, S. L., Burns, D. S., Docherty, S. L., & Haase, J. E. (2011). Ensuring treatment fidelity in a multi-site behavioral intervention study: Implementing NIH Behavior Change Consortium recommendations in the smart trial. *Psycho-Oncology*, *20*, 1193–1201. doi: 10.1002/pon.1845 22012943 PMC3198011

[CIT0075] Roy, R., Colquhoun, H., Byrne, M., Lorencatto, F., Matvienko-Sikar, K., Mccleary, N., … Toomey, E. (2018). Addressing fidelity within complex health behaviour change interventions: A protocol of a scoping review of intervention fidelity frameworks and models. [version 1; peer review: 2 approved]. *HRB Open Research*, *1*(25). doi:10.12688/hrbopenres.12892.1 10.1080/17437199.2025.2534001 40744005

[CIT0076] Schinckus, L., Van Den Broucke, S., & Housiaux, M. (2014). Assessment of implementation fidelity in diabetes self-management education programs: A systematic review. *Patient Education and Counseling*, *96*, 13–21. doi: 10.1016/j.pec.2014.04.002 24795074

[CIT0077] Schober, I., Sharpe, H., & Schmidt, U. (2013). The reporting of fidelity measures in primary prevention programmes for eating disorders in schools. *European Eating Disorders Review*, *21*, 374–381. doi: 10.1002/erv.2243 23881537

[CIT0078] Schulz, K. F., Altman, D. G., & Moher, D. (2010). Consort 2010 statement: Updated guidelines for reporting parallel group randomised trials. *BMJ*, *340*, C332. doi: 10.1136/bmj.c332 20332509 PMC2844940

[CIT0079] Shaw, R. L., Hiles, D. R., West, K., Holland, C., & Gwyther, H. (2018). From mixing methods to the logic(s) of inquiry: Taking a fresh look at developing mixed design studies. *Health Psychology And Behavioral Medicine*, *6*, 226–244. doi: 10.1080/21642850.2018.1515016 PMC8114392 34040830

[CIT0080] Slaughter, S. E., Hill, J. N., & Snelgrove-Clarke, E. (2015). What is the extent and quality of documentation and reporting of fidelity to implementation strategies: A scoping review. *Implementation Science*, *10*, 129–129. doi: 10.1186/s13012-015-0320-3 26345357 PMC4562107

[CIT0081] Spillane, V., Byrne, M. C., Byrne, M., Leathem, C. S., O’Malley, M., & Cupples, M. E. (2007). Monitoring treatment fidelity in a randomized controlled trial of a complex intervention. *Journal of Advanced Nursing*, *60*, 343–352. doi: 10.1111/j.1365-2648.2007.04386.x 17908130

[CIT0082] Sullivan-Bolyai, S., Crawford, S., Bova, C., Lee, M., Quintos, J. B., Johnson, K., … Melkus, G. (2015). PETS-D: Impact on diabetes management outcomes. *The Diabetes Educator*, *41*, 537–549. doi: 10.1177/0145721715598383 26246593

[CIT0083] Thompson, T. P., Lambert, J. D., Greaves, C. J., & Taylor, A. H. (2018). Intervention delivery fidelity assessment of a counseling-based intervention for promoting smoking reduction and increasing physical activity. *Health Psychology*, *37*, 627–637. doi: 10.1037/hea0000613 29708387

[CIT0084] Toomey, E., Currie-Murphy, L., Matthews, J., & Hurley, D. A. (2014). Implementation fidelity of physiotherapist-delivered group education and exercise interventions to promote self-management in people with osteoarthritis and chronic low back pain: A rapid review part II. *Manual Therapy*, *20*, 287–294. doi: 10.1016/j.math.2014.10.012 25466294

[CIT0085] Toomey, E., & Hardeman, W. (2017). Addressing intervention fidelity within physical therapy research and clinical practice. *Journal of Orthopaedic Sports Physical Therapy*, *47*, 895–898. doi: 10.2519/jospt.2017.0609 29191117

[CIT0086] Toomey, E., Matthews, J., Guerin, S., & Hurley, D. A. (2016). Development of a feasible implementation fidelity protocol within a complex physical therapy-led self-management intervention. *Physical Therapy*, *96*, 1287–1298. doi: 10.2522/ptj.20150446 26939605

[CIT0087] Toomey, E., Matthews, J., & Hurley, D. A. (2017). Using mixed methods to assess fidelity of delivery and its influencing factors in a complex self-management intervention for people with osteoarthritis and low back pain. *BMJ Open*, *7*, e015452. 10.1136/bmjopen-2016-015452 PMC5724095 28780544

[CIT0088] Toomey, E., Matvienko-Sikar, K., Heary, C., Delaney, L., Queally, M., Hayes, C. B., … Choosing Healthy Eating for Infant Health (CHErIsH) study team. (2018). Intervention fidelity within trials of infant feeding behavioral interventions to prevent childhood obesity: A systematic review. *Annals of Behavioral Medicine*, *53*(1), 75–97. doi: 10.1093/abm/kay021 29796664

[CIT0089] Walton, H., Spector, A., Tombor, I., & Michie, S. (2017). Measures of fidelity of delivery of, and engagement with, complex, face-to-face health behaviour change interventions: A systematic review of measure quality. *British Journal of Health Psychology*, *22*, 872–903. doi: 10.1111/bjhp.12260 28762607 PMC5655766

[CIT0090] Waltz, J., Addis, M. E., Koerner, K., & Jacobson, N. S. (1993). Testing the integrity of a psychotherapy protocol: Assessment of adherence and competence. *Journal of Consulting and Clinical Psychology*, *61*, 620–630. doi: 10.1037/0022-006X.61.4.620 8370857

[CIT0091] Weiner, B. J., Lewis, C. C., Stanick, C., Powell, B. J., Dorsey, C. N., Clary, A. S., … Halko, H. (2017). Psychometric assessment of three newly developed implementation outcome measures. *Implementation Science*, *12*, 108. doi: 10.1186/s13012-017-0635-3 28851459 PMC5576104

[CIT0092] Williams, S. L., Mcsharry, J., Taylor, C., Dale, J., Michie, S., & French, D. P. (2020). Translating a walking intervention for health professional delivery within primary care: A mixed-methods treatment fidelity assessment. *British Journal Of Health Psychology*, *25*, 17–38. doi: 10.1111/bjhp.12392 31746091 PMC7003875

[CIT0093] Yamato, T. P., Maher, C. G., Saragiotto, B. T., Hoffmann, T. C., & Moseley, A. M. (2016). How completely are physiotherapy interventions described in reports of randomised trials? *Physiotherapy*, *102*, 121–126. doi: 10.1016/j.physio.2016.03.001 27033780

[CIT0094] Yeaton, W., & Sechrest, L. (1981). Critical dimensions in the choice and maintenance of successful treatments: Strength, integrity, and effectiveness. *Journal of Consulting and Clinical Psychology*, *49*(2), 156–167. 10.1037//0022-006X.49.2.156 doi: 10.1037//0022-006X.49.2.156 7217482

[CIT0095] Yu, A. M., Balasubramanaiam, B., Offringa, M., & Kelly, L. E. (2018). Reporting of interventions and “standard of care” control arms in pediatric clinical trials: A quantitative analysis. *Pediatric Research*, *84*, 393–398. doi: 10.1038/s41390-018-0019-7 29899384

